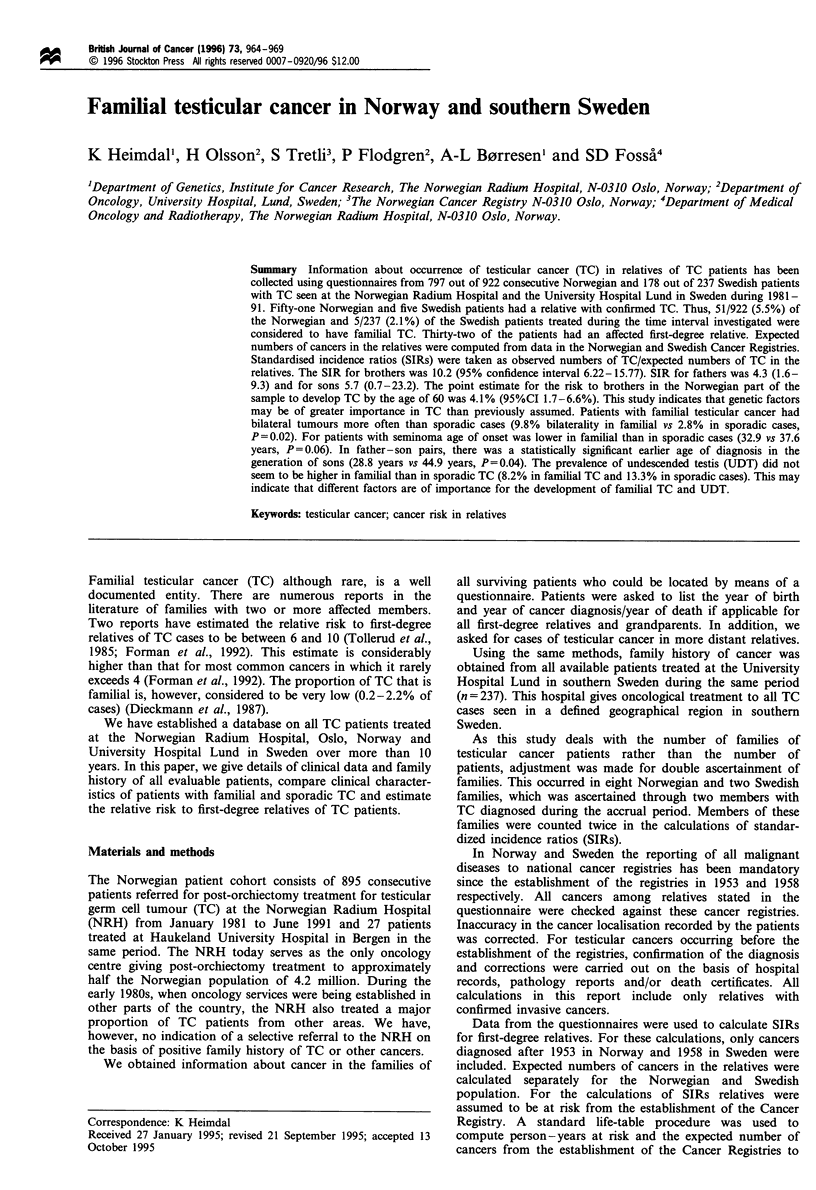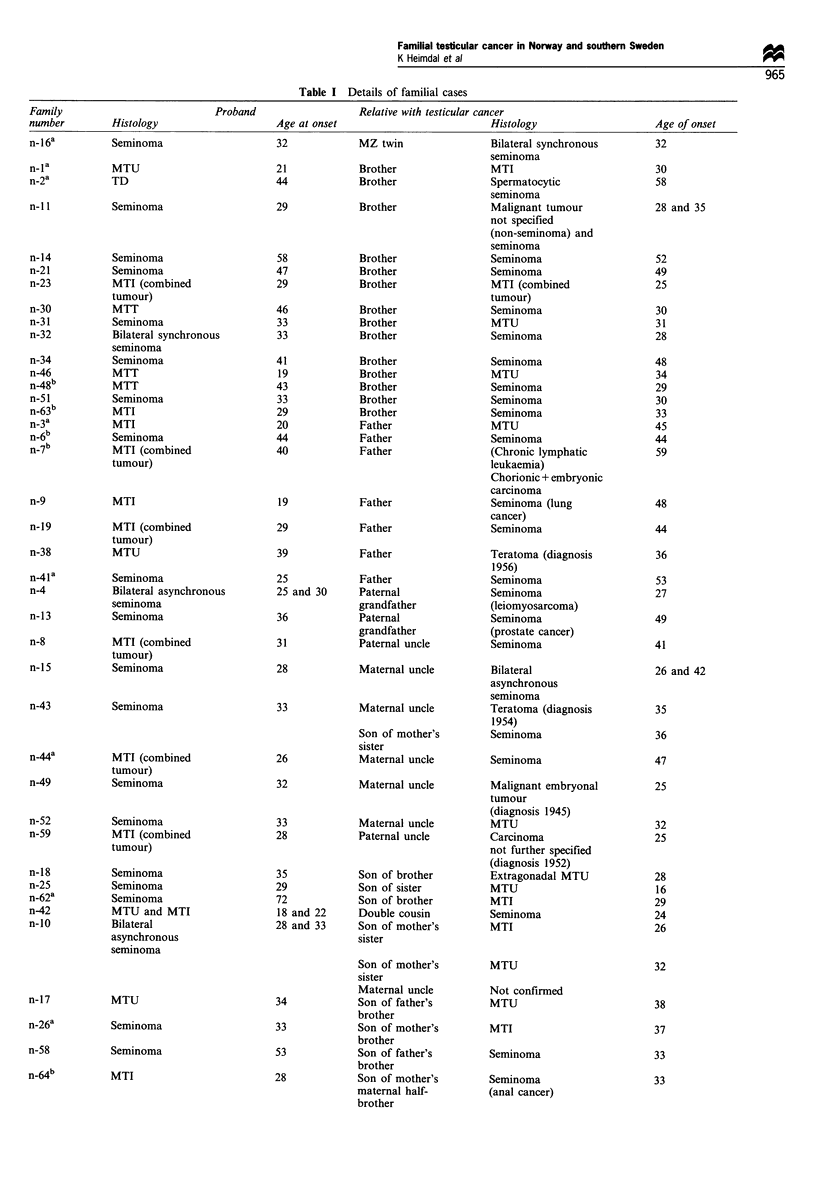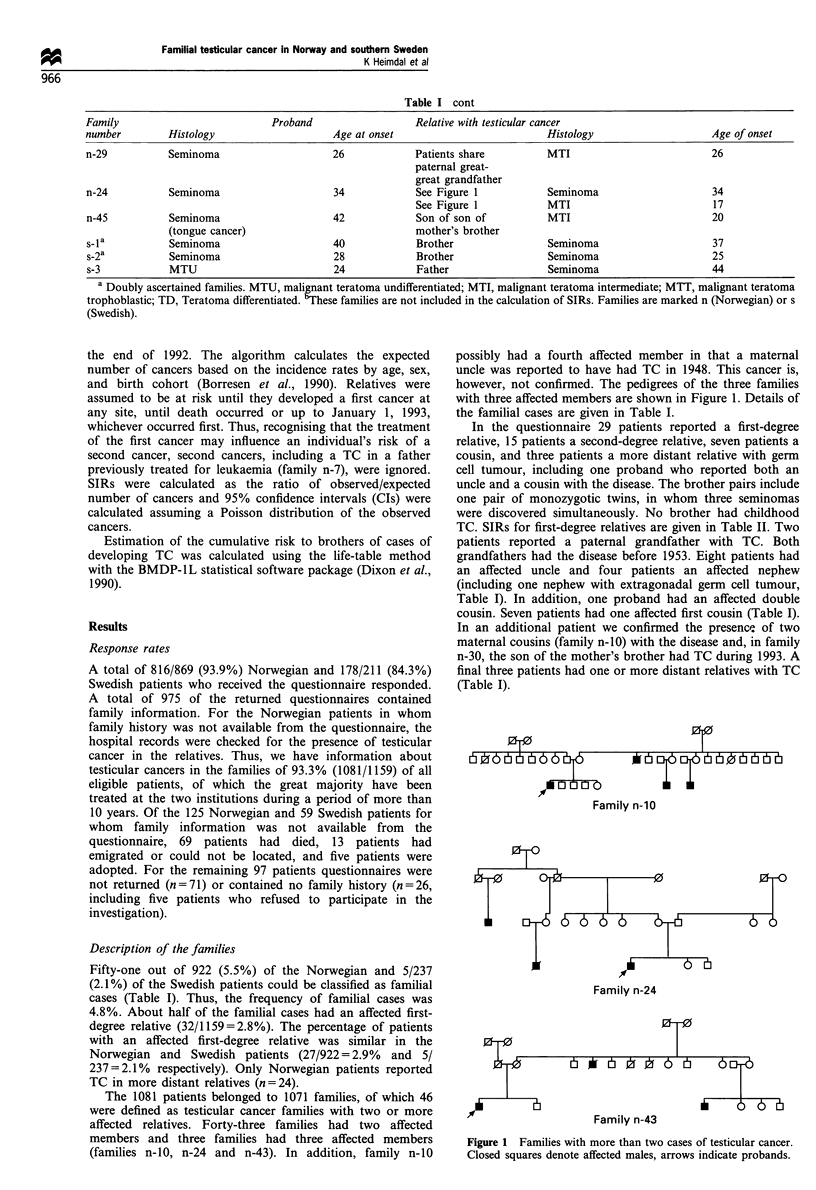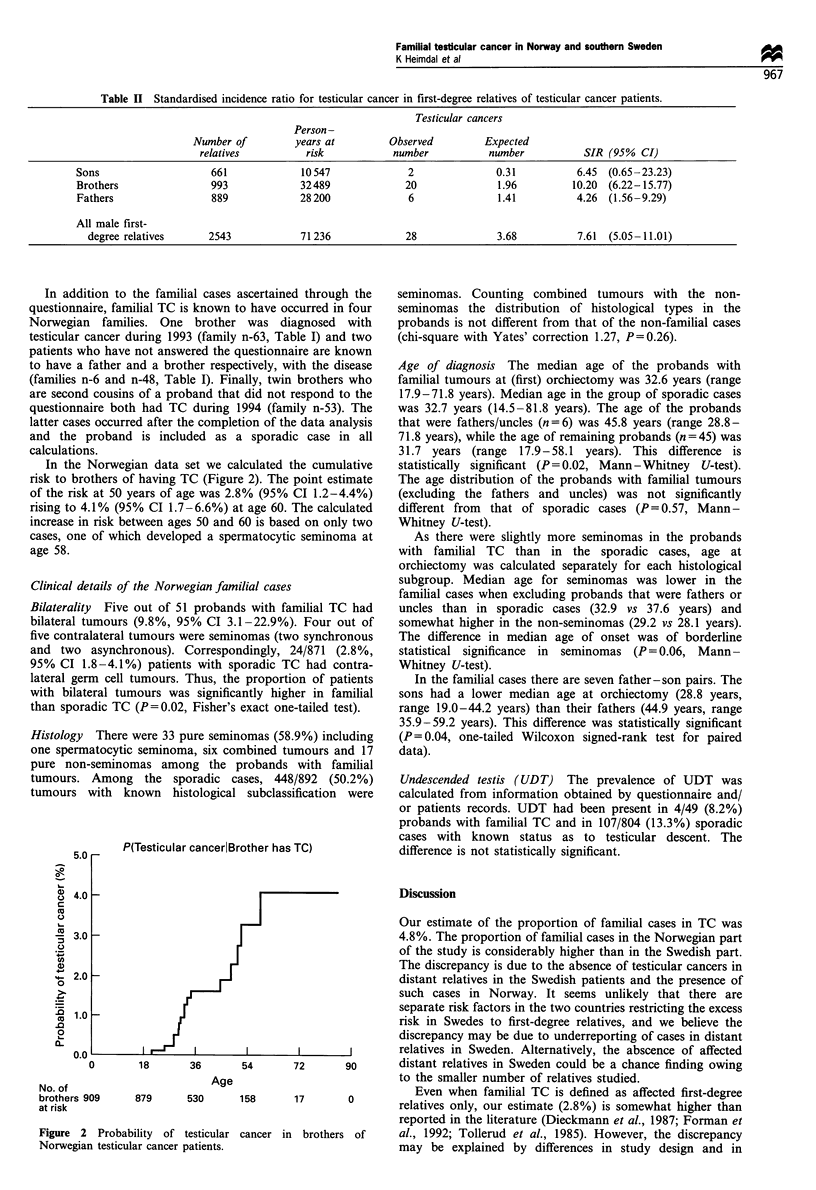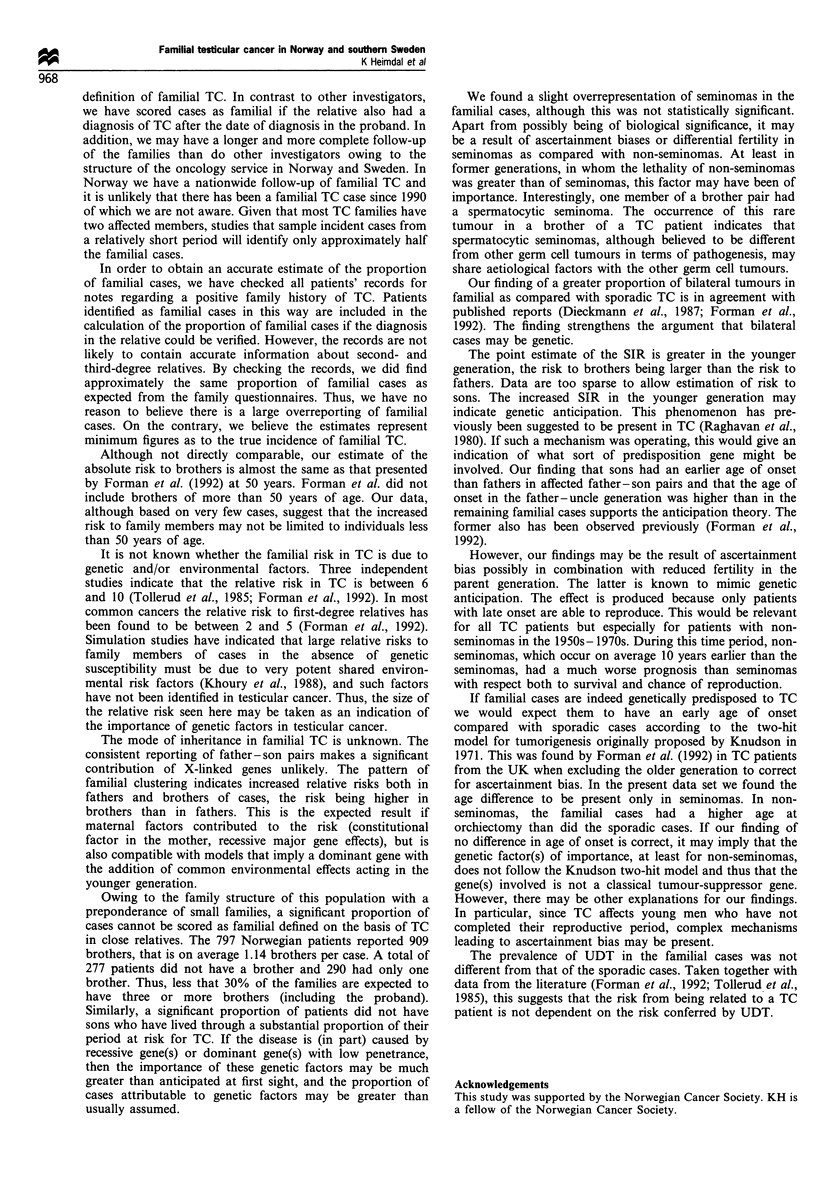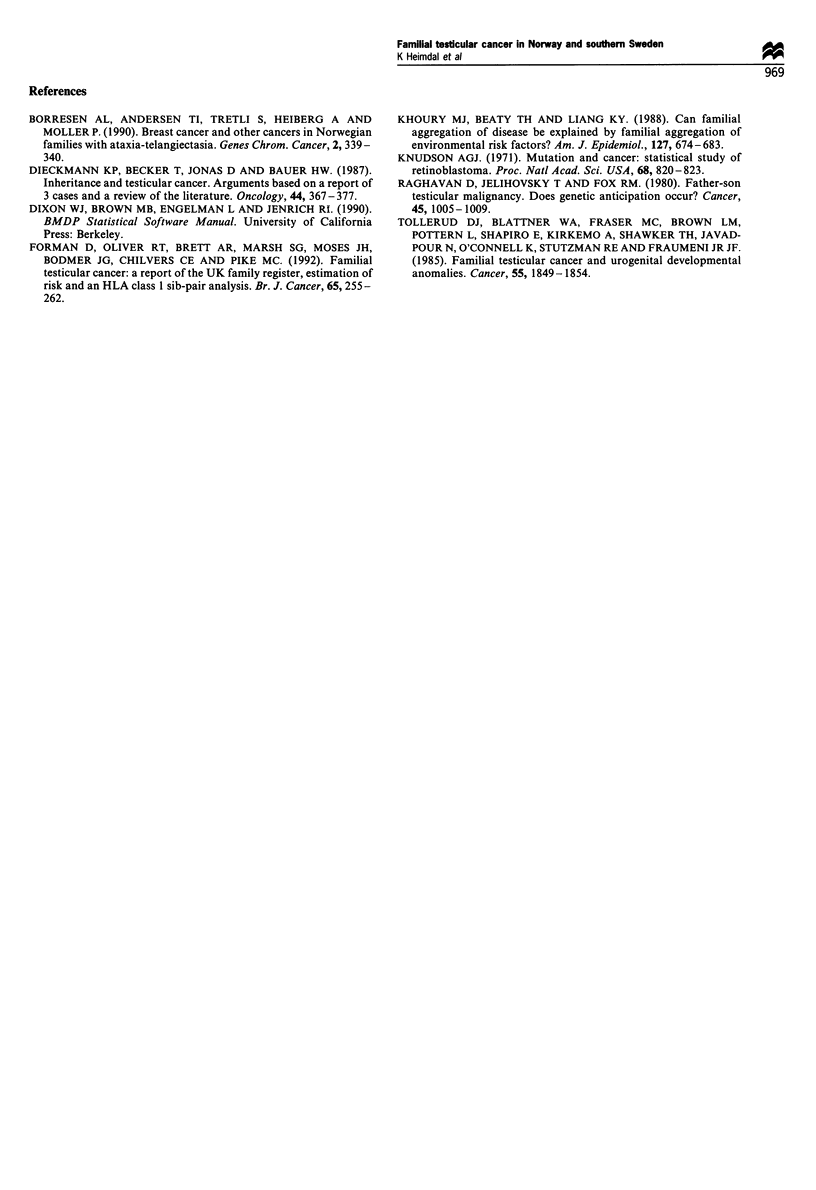# Familial testicular cancer in Norway and southern Sweden.

**DOI:** 10.1038/bjc.1996.173

**Published:** 1996-04

**Authors:** K. Heimdal, H. Olsson, S. Tretli, P. Flodgren, A. L. Børresen, S. D. Fossa

**Affiliations:** Department of Genetics, Institute for Cancer Research, The Norwegian Radium Hospital, Oslo, Norway.

## Abstract

Information about occurrence of testicular cancer (TC) in relatives of TC patients has been collected using questionnaires from 797 out of 922 consecutive Norwegian and 178 out of 237 Swedish patients with TC seen at the Norwegian Radium Hospital and the University Hospital Lund in Sweden during 1981-91. Fifty-one Norwegian and five Swedish patients had a relative with confirmed TC. Thus, 51/922 (5.5%) of the Norwegian and 5/237 (2.1%) of the Swedish patients treated during the time interval investigated were considered to have familial TC. Thirty-two of the patients had an affected first-degree relative. Expected numbers of cancers in the relatives were computed from data in the Norwegian and Swedish Cancer Registries. Standardised incidence ratios (SIRs) were taken as observed numbers of TC/expected numbers of TC in the relatives. The SIR for brothers was 10.2 (95% confidence interval 6.22-15.77). SIR for fathers was 4.3 (1.6-9.3) and for sons 5.7 (0.7-23.2). The point estimate for the risk to brothers in the Norwegian part of the sample to develop TC by the age of 60 was 4.1% (95% CI 1.7-6.6%). This study indicates that genetic factors may be of greater importance in TC than previously assumed. Patients with familial testicular cancer had bilateral tumours more often than sporadic cases (9.8% bilaterality in familial vs 2.8% in sporadic cases, P=0.02). For patients with seminoma age of onset was lower in familial than in sporadic cases (32.9 vs 37.6 years, P=0.06). In father-son pairs, there was a statistically significant earlier age of diagnosis in the generation of sons (28.8 years vs 44.9 years, P=0.04). The prevalence of undescended testis (UDT) did not seem to be higher in familial than in sporadic TC (8.2% in familial TC and 13.3% in sporadic cases). This may indicate that different factors are of importance for the development of familial TC and UDT.


British Journal of Cancer (1996) 73, 964–969
© 1996 Stockton Press All rights reserved 0007-0920/96 $12.00

### Familial testicular cancer in Norway and southern Sweden

K Heimdal[1], H Olsson[2], S Tretli[3], P Flodgren[2], A-L Børresen[1] and SD Fosså[4]

[1]*Department of Genetics, Institute for Cancer Research, The Norwegian Radium Hospital, N-0310 Oslo, Norway;* [2]*Department of Oncology, University Hospital, Lund, Sweden;* [3]*The Norwegian Cancer Registry N-0310 Oslo, Norway;* [4]*Department of Medical Oncology and Radiotherapy, The Norwegian Radium Hospital, N-0310 Oslo, Norway.*

**Summary** Information about occurrence of testicular cancer (TC) in relatives of TC patients has been collected using questionnaires from 797 out of 922 consecutive Norwegian and 178 out of 237 Swedish patients with TC seen at the Norwegian Radium Hospital and the University Hospital Lund in Sweden during 1981–91. Fifty-one Norwegian and five Swedish patients had a relative with confirmed TC. Thus, 51/922 (5.5%) of the Norwegian and 5/237 (2.1%) of the Swedish patients treated during the time interval investigated were considered to have familial TC. Thirty-two of the patients had an affected first-degree relative. Expected numbers of cancers in the relatives were computed from data in the Norwegian and Swedish Cancer Registries. Standardised incidence ratios (SIRs) were taken as observed numbers of TC/expected numbers of TC in the relatives. The SIR for brothers was 10.2 (95% confidence interval 6.22–15.77). SIR for fathers was 4.3 (1.6–9.3) and for sons 5.7 (0.7–23.2). The point estimate for the risk to brothers in the Norwegian part of the sample to develop TC by the age of 60 was 4.1% (95%CI 1.7–6.6%). This study indicates that genetic factors may be of greater importance in TC than previously assumed. Patients with familial testicular cancer had bilateral tumours more often than sporadic cases (9.8% bilaterality in familial *vs* 2.8% in sporadic cases, $P = 0.02$). For patients with seminoma age of onset was lower in familial than in sporadic cases (32.9 *vs* 37.6 years, $P = 0.06$). In father–son pairs, there was a statistically significant earlier age of diagnosis in the generation of sons (28.8 years *vs* 44.9 years, $P = 0.04$). The prevalence of undescended testis (UDT) did not seem to be higher in familial than in sporadic TC (8.2% in familial TC and 13.3% in sporadic cases). This may indicate that different factors are of importance for the development of familial TC and UDT.

**Keywords:** testicular cancer; cancer risk in relatives

Familial testicular cancer (TC) although rare, is a well documented entity. There are numerous reports in the literature of families with two or more affected members. Two reports have estimated the relative risk to first-degree relatives of TC cases to be between 6 and 10 (Tollerud *et al.*, 1985; Forman *et al.*, 1992). This estimate is considerably higher than that for most common cancers in which it rarely exceeds 4 (Forman *et al.*, 1992). The proportion of TC that is familial is, however, considered to be very low (0.2–2.2% of cases) (Dieckmann *et al.*, 1987).

We have established a database on all TC patients treated at the Norwegian Radium Hospital, Oslo, Norway and University Hospital Lund in Sweden over more than 10 years. In this paper, we give details of clinical data and family history of all evaluable patients, compare clinical characteristics of patients with familial and sporadic TC and estimate the relative risk to first-degree relatives of TC patients.

### Materials and methods

The Norwegian patient cohort consists of 895 consecutive patients referred for post-orchiectomy treatment for testicular germ cell tumour (TC) at the Norwegian Radium Hospital (NRH) from January 1981 to June 1991 and 27 patients treated at Haukeland University Hospital in Bergen in the same period. The NRH today serves as the only oncology centre giving post-orchiectomy treatment to approximately half the Norwegian population of 4.2 million. During the early 1980s, when oncology services were being established in other parts of the country, the NRH also treated a major proportion of TC patients from other areas. We have, however, no indication of a selective referral to the NRH on the basis of positive family history of TC or other cancers.

We obtained information about cancer in the families of all surviving patients who could be located by means of a questionnaire. Patients were asked to list the year of birth and year of cancer diagnosis/year of death if applicable for all first-degree relatives and grandparents. In addition, we asked for cases of testicular cancer in more distant relatives.

Using the same methods, family history of cancer was obtained from all available patients treated at the University Hospital Lund in southern Sweden during the same period ($n = 237$). This hospital gives oncological treatment to all TC cases seen in a defined geographical region in southern Sweden.

As this study deals with the number of families of testicular cancer patients rather than the number of patients, adjustment was made for double ascertainment of families. This occurred in eight Norwegian and two Swedish families, which was ascertained through two members with TC diagnosed during the accrual period. Members of these families were counted twice in the calculations of standardized incidence ratios (SIRs).

In Norway and Sweden the reporting of all malignant diseases to national cancer registries has been mandatory since the establishment of the registries in 1953 and 1958 respectively. All cancers among relatives stated in the questionnaire were checked against these cancer registries. Inaccuracy in the cancer localisation recorded by the patients was corrected. For testicular cancers occurring before the establishment of the registries, confirmation of the diagnosis and corrections were carried out on the basis of hospital records, pathology reports and/or death certificates. All calculations in this report include only relatives with confirmed invasive cancers.

Data from the questionnaires were used to calculate SIRs for first-degree relatives. For these calculations, only cancers diagnosed after 1953 in Norway and 1958 in Sweden were included. Expected numbers of cancers in the relatives were calculated separately for the Norwegian and Swedish population. For the calculations of SIRs relatives were assumed to be at risk from the establishment of the Cancer Registry. A standard life-table procedure was used to compute person–years at risk and the expected number of cancers from the establishment of the Cancer Registries to

Correspondence: K Heimdal
Received 27 January 1995; revised 21 September 1995; accepted 13 October 1995

**Table I** Details of familial cases

| *Family number* | *Proband* *Histology* | *Age at onset* | *Relative with testicular cancer* | *Histology* | *Age of onset* |
|---|---|---|---|---|---|
| n-16[a] | Seminoma | 32 | MZ twin | Bilateral synchronous seminoma | 32 |
| n-1[a] | MTU | 21 | Brother | MTI | 30 |
| n-2[a] | TD | 44 | Brother | Spermatocytic seminoma | 58 |
| n-11 | Seminoma | 29 | Brother | Malignant tumour not specified (non-seminoma) and seminoma | 28 and 35 |
| n-14 | Seminoma | 58 | Brother | Seminoma | 52 |
| n-21 | Seminoma | 47 | Brother | Seminoma | 49 |
| n-23 | MTI (combined tumour) | 29 | Brother | MTI (combined tumour) | 25 |
| n-30 | MTT | 46 | Brother | Seminoma | 30 |
| n-31 | Seminoma | 33 | Brother | MTU | 31 |
| n-32 | Bilateral synchronous seminoma | 33 | Brother | Seminoma | 28 |
| n-34 | Seminoma | 41 | Brother | Seminoma | 48 |
| n-46 | MTT | 19 | Brother | MTU | 34 |
| n-48[b] | MTT | 43 | Brother | Seminoma | 29 |
| n-51 | Seminoma | 33 | Brother | Seminoma | 30 |
| n-63[b] | MTI | 29 | Brother | Seminoma | 33 |
| n-3[a] | MTI | 20 | Father | MTU | 45 |
| n-6[b] | Seminoma | 44 | Father | Seminoma | 44 |
| n-7[b] | MTI (combined tumour) | 40 | Father | (Chronic lymphatic leukaemia) Chorionic + embryonic carcinoma | 59 |
| n-9 | MTI | 19 | Father | Seminoma (lung cancer) | 48 |
| n-19 | MTI (combined tumour) | 29 | Father | Seminoma | 44 |
| n-38 | MTU | 39 | Father | Teratoma (diagnosis 1956) | 36 |
| n-41[a] | Seminoma | 25 | Father | Seminoma | 53 |
| n-4 | Bilateral asynchronous seminoma | 25 and 30 | Paternal grandfather | Seminoma (leiomyosarcoma) | 27 |
| n-13 | Seminoma | 36 | Paternal grandfather | Seminoma (prostate cancer) | 49 |
| n-8 | MTI (combined tumour) | 31 | Paternal uncle | Seminoma | 41 |
| n-15 | Seminoma | 28 | Maternal uncle | Bilateral asynchronous seminoma | 26 and 42 |
| n-43 | Seminoma | 33 | Maternal uncle | Teratoma (diagnosis 1954) | 35 |
| | | | Son of mother's sister | Seminoma | 36 |
| n-44[a] | MTI (combined tumour) | 26 | Maternal uncle | Seminoma | 47 |
| n-49 | Seminoma | 32 | Maternal uncle | Malignant embryonal tumour (diagnosis 1945) | 25 |
| n-52 | Seminoma | 33 | Maternal uncle | MTU | 32 |
| n-59 | MTI (combined tumour) | 28 | Paternal uncle | Carcinoma not further specified (diagnosis 1952) | 25 |
| n-18 | Seminoma | 35 | Son of brother | Extragonadal MTU | 28 |
| n-25 | Seminoma | 29 | Son of sister | MTU | 16 |
| n-62[a] | Seminoma | 72 | Son of brother | MTI | 29 |
| n-42 | MTU and MTI | 18 and 22 | Double cousin | Seminoma | 24 |
| n-10 | Bilateral asynchronous seminoma | 28 and 33 | Son of mother's sister | MTI | 26 |
| | | | Son of mother's sister | MTU | 32 |
| | | | Maternal uncle | Not confirmed | |
| n-17 | MTU | 34 | Son of father's brother | MTU | 38 |
| n-26[a] | Seminoma | 33 | Son of mother's brother | MTI | 37 |
| n-58 | Seminoma | 53 | Son of father's brother | Seminoma | 33 |
| n-64[b] | MTI | 28 | Son of mother's maternal half-brother | Seminoma (anal cancer) | 33 |

Table I cont

| Family number | Proband Histology | Age at onset | Relative with testicular cancer | Histology | Age of onset |
|---|---|---|---|---|---|
| n-29 | Seminoma | 26 | Patients share paternal great-great grandfather | MTI | 26 |
| n-24 | Seminoma | 34 | See Figure 1 | Seminoma | 34 |
| | | | See Figure 1 | MTI | 17 |
| n-45 | Seminoma (tongue cancer) | 42 | Son of son of mother's brother | MTI | 20 |
| s-1[a] | Seminoma | 40 | Brother | Seminoma | 37 |
| s-2[a] | Seminoma | 28 | Brother | Seminoma | 25 |
| s-3 | MTU | 24 | Father | Seminoma | 44 |

[a] Doubly ascertained families. MTU, malignant teratoma undifferentiated; MTI, malignant teratoma intermediate; MTT, malignant teratoma trophoblastic; TD, Teratoma differentiated. [b]These families are not included in the calculation of SIRs. Families are marked n (Norwegian) or s (Swedish).

the end of 1992. The algorithm calculates the expected number of cancers based on the incidence rates by age, sex, and birth cohort (Borresen *et al.*, 1990). Relatives were assumed to be at risk until they developed a first cancer at any site, until death occurred or up to January 1, 1993, whichever occurred first. Thus, recognising that the treatment of the first cancer may influence an individual's risk of a second cancer, second cancers, including a TC in a father previously treated for leukaemia (family n-7), were ignored. SIRs were calculated as the ratio of observed/expected number of cancers and 95% confidence intervals (CIs) were calculated assuming a Poisson distribution of the observed cancers.

Estimation of the cumulative risk to brothers of cases of developing TC was calculated using the life-table method with the BMDP-1L statistical software package (Dixon *et al.*, 1990).

### Results

*Response rates*

A total of 816/869 (93.9%) Norwegian and 178/211 (84.3%) Swedish patients who received the questionnaire responded. A total of 975 of the returned questionnaires contained family information. For the Norwegian patients in whom family history was not available from the questionnaire, the hospital records were checked for the presence of testicular cancer in the relatives. Thus, we have information about testicular cancers in the families of 93.3% (1081/1159) of all eligible patients, of which the great majority have been treated at the two institutions during a period of more than 10 years. Of the 125 Norwegian and 59 Swedish patients for whom family information was not available from the questionnaire, 69 patients had died, 13 patients had emigrated or could not be located, and five patients were adopted. For the remaining 97 patients questionnaires were not returned ($n=71$) or contained no family history ($n=26$, including five patients who refused to participate in the investigation).

*Description of the families*

Fifty-one out of 922 (5.5%) of the Norwegian and 5/237 (2.1%) of the Swedish patients could be classified as familial cases (Table I). Thus, the frequency of familial cases was 4.8%. About half of the familial cases had an affected first-degree relative (32/1159 = 2.8%). The percentage of patients with an affected first-degree relative was similar in the Norwegian and Swedish patients (27/922 = 2.9% and 5/237 = 2.1% respectively). Only Norwegian patients reported TC in more distant relatives ($n=24$).

The 1081 patients belonged to 1071 families, of which 46 were defined as testicular cancer families with two or more affected relatives. Forty-three families had two affected members and three families had three affected members (families n-10, n-24 and n-43). In addition, family n-10 possibly had a fourth affected member in that a maternal uncle was reported to have had TC in 1948. This cancer is, however, not confirmed. The pedigrees of the three families with three affected members are shown in Figure 1. Details of the familial cases are given in Table I.

In the questionnaire 29 patients reported a first-degree relative, 15 patients a second-degree relative, seven patients a cousin, and three patients a more distant relative with germ cell tumour, including one proband who reported both an uncle and a cousin with the disease. The brother pairs include one pair of monozygotic twins, in whom three seminomas were discovered simultaneously. No brother had childhood TC. SIRs for first-degree relatives are given in Table II. Two patients reported a paternal grandfather with TC. Both grandfathers had the disease before 1953. Eight patients had an affected uncle and four patients an affected nephew (including one nephew with extragonadal germ cell tumour, Table I). In addition, one proband had an affected double cousin. Seven patients had one affected first cousin (Table I). In an additional patient we confirmed the presence of two maternal cousins (family n-10) with the disease and, in family n-30, the son of the mother's brother had TC during 1993. A final three patients had one or more distant relatives with TC (Table I).

**Figure 1** Families with more than two cases of testicular cancer. Closed squares denote affected males, arrows indicate probands.

**Table II** Standardised incidence ratio for testicular cancer in first-degree relatives of testicular cancer patients.

| | *Number of relatives* | *Person–years at risk* | *Testicular cancers Observed number* | *Expected number* | *SIR (95% CI)* |
|---|---|---|---|---|---|
| Sons | 661 | 10 547 | 2 | 0.31 | 6.45 (0.65–23.23) |
| Brothers | 993 | 32 489 | 20 | 1.96 | 10.20 (6.22–15.77) |
| Fathers | 889 | 28 200 | 6 | 1.41 | 4.26 (1.56–9.29) |
| All male first-degree relatives | 2543 | 71 236 | 28 | 3.68 | 7.61 (5.05–11.01) |

In addition to the familial cases ascertained through the questionnaire, familial TC is known to have occurred in four Norwegian families. One brother was diagnosed with testicular cancer during 1993 (family n-63, Table I) and two patients who have not answered the questionnaire are known to have a father and a brother respectively, with the disease (families n-6 and n-48, Table I). Finally, twin brothers who are second cousins of a proband that did not respond to the questionnaire both had TC during 1994 (family n-53). The latter cases occurred after the completion of the data analysis and the proband is included as a sporadic case in all calculations.

In the Norwegian data set we calculated the cumulative risk to brothers of having TC (Figure 2). The point estimate of the risk at 50 years of age was 2.8% (95% CI 1.2–4.4%) rising to 4.1% (95% CI 1.7–6.6%) at age 60. The calculated increase in risk between ages 50 and 60 is based on only two cases, one of which developed a spermatocytic seminoma at age 58.

### *Clinical details of the Norwegian familial cases*

*Bilaterality* Five out of 51 probands with familial TC had bilateral tumours (9.8%, 95% CI 3.1–22.9%). Four out of five contralateral tumours were seminomas (two synchronous and two asynchronous). Correspondingly, 24/871 (2.8%, 95% CI 1.8–4.1%) patients with sporadic TC had contralateral germ cell tumours. Thus, the proportion of patients with bilateral tumours was significantly higher in familial than sporadic TC ($P = 0.02$, Fisher's exact one-tailed test).

*Histology* There were 33 pure seminomas (58.9%) including one spermatocytic seminoma, six combined tumours and 17 pure non-seminomas among the probands with familial tumours. Among the sporadic cases, 448/892 (50.2%) tumours with known histological subclassification were

**Figure 2** Probability of testicular cancer in brothers of Norwegian testicular cancer patients.

seminomas. Counting combined tumours with the non-seminomas the distribution of histological types in the probands is not different from that of the non-familial cases (chi-square with Yates' correction 1.27, $P = 0.26$).

*Age of diagnosis* The median age of the probands with familial tumours at (first) orchiectomy was 32.6 years (range 17.9–71.8 years). Median age in the group of sporadic cases was 32.7 years (14.5–81.8 years). The age of the probands that were fathers/uncles ($n = 6$) was 45.8 years (range 28.8–71.8 years), while the age of remaining probands ($n = 45$) was 31.7 years (range 17.9–58.1 years). This difference is statistically significant ($P = 0.02$, Mann–Whitney $U$-test). The age distribution of the probands with familial tumours (excluding the fathers and uncles) was not significantly different from that of sporadic cases ($P = 0.57$, Mann–Whitney $U$-test).

As there were slightly more seminomas in the probands with familial TC than in the sporadic cases, age at orchiectomy was calculated separately for each histological subgroup. Median age for seminomas was lower in the familial cases when excluding probands that were fathers or uncles than in sporadic cases (32.9 *vs* 37.6 years) and somewhat higher in the non-seminomas (29.2 *vs* 28.1 years). The difference in median age of onset was of borderline statistical significance in seminomas ($P = 0.06$, Mann–Whitney $U$-test).

In the familial cases there are seven father–son pairs. The sons had a lower median age at orchiectomy (28.8 years, range 19.0–44.2 years) than their fathers (44.9 years, range 35.9–59.2 years). This difference was statistically significant ($P = 0.04$, one-tailed Wilcoxon signed-rank test for paired data).

*Undescended testis (UDT)* The prevalence of UDT was calculated from information obtained by questionnaire and/or patients records. UDT had been present in 4/49 (8.2%) probands with familial TC and in 107/804 (13.3%) sporadic cases with known status as to testicular descent. The difference is not statistically significant.

### Discussion

Our estimate of the proportion of familial cases in TC was 4.8%. The proportion of familial cases in the Norwegian part of the study is considerably higher than in the Swedish part. The discrepancy is due to the absence of testicular cancers in distant relatives in the Swedish patients and the presence of such cases in Norway. It seems unlikely that there are separate risk factors in the two countries restricting the excess risk in Swedes to first-degree relatives, and we believe the discrepancy may be due to underreporting of cases in distant relatives in Sweden. Alternatively, the abscence of affected distant relatives in Sweden could be a chance finding owing to the smaller number of relatives studied.

Even when familial TC is defined as affected first-degree relatives only, our estimate (2.8%) is somewhat higher than reported in the literature (Dieckmann *et al.*, 1987; Forman *et al.*, 1992; Tollerud *et al.*, 1985). However, the discrepancy may be explained by differences in study design and in definition of familial TC. In contrast to other investigators, we have scored cases as familial if the relative also had a diagnosis of TC after the date of diagnosis in the proband. In addition, we may have a longer and more complete follow-up of the families than do other investigators owing to the structure of the oncology service in Norway and Sweden. In Norway we have a nationwide follow-up of familial TC and it is unlikely that there has been a familial TC case since 1990 of which we are not aware. Given that most TC families have two affected members, studies that sample incident cases from a relatively short period will identify only approximately half the familial cases.

In order to obtain an accurate estimate of the proportion of familial cases, we have checked all patients' records for notes regarding a positive family history of TC. Patients identified as familial cases in this way are included in the calculation of the proportion of familial cases if the diagnosis in the relative could be verified. However, the records are not likely to contain accurate information about second- and third-degree relatives. By checking the records, we did find approximately the same proportion of familial cases as expected from the family questionnaires. Thus, we have no reason to believe there is a large overreporting of familial cases. On the contrary, we believe the estimates represent minimum figures as to the true incidence of familial TC.

Although not directly comparable, our estimate of the absolute risk to brothers is almost the same as that presented by Forman *et al.* (1992) at 50 years. Forman *et al.* did not include brothers of more than 50 years of age. Our data, although based on very few cases, suggest that the increased risk to family members may not be limited to individuals less than 50 years of age.

It is not known whether the familial risk in TC is due to genetic and/or environmental factors. Three independent studies indicate that the relative risk in TC is between 6 and 10 (Tollerud *et al.*, 1985; Forman *et al.*, 1992). In most common cancers the relative risk to first-degree relatives has been found to be between 2 and 5 (Forman *et al.*, 1992). Simulation studies have indicated that large relative risks to family members of cases in the absence of genetic susceptibility must be due to very potent shared environmental risk factors (Khoury *et al.*, 1988), and such factors have not been identified in testicular cancer. Thus, the size of the relative risk seen here may be taken as an indication of the importance of genetic factors in testicular cancer.

The mode of inheritance in familial TC is unknown. The consistent reporting of father–son pairs makes a significant contribution of X-linked genes unlikely. The pattern of familial clustering indicates increased relative risks both in fathers and brothers of cases, the risk being higher in brothers than in fathers. This is the expected result if maternal factors contributed to the risk (constitutional factor in the mother, recessive major gene effects), but is also compatible with models that imply a dominant gene with the addition of common environmental effects acting in the younger generation.

Owing to the family structure of this population with a preponderance of small families, a significant proportion of cases cannot be scored as familial defined on the basis of TC in close relatives. The 797 Norwegian patients reported 909 brothers, that is on average 1.14 brothers per case. A total of 277 patients did not have a brother and 290 had only one brother. Thus, less that 30% of the families are expected to have three or more brothers (including the proband). Similarly, a significant proportion of patients did not have sons who have lived through a substantial proportion of their period at risk for TC. If the disease is (in part) caused by recessive gene(s) or dominant gene(s) with low penetrance, then the importance of these genetic factors may be much greater than anticipated at first sight, and the proportion of cases attributable to genetic factors may be greater than usually assumed.

We found a slight overrepresentation of seminomas in the familial cases, although this was not statistically significant. Apart from possibly being of biological significance, it may be a result of ascertainment biases or differential fertility in seminomas as compared with non-seminomas. At least in former generations, in whom the lethality of non-seminomas was greater than of seminomas, this factor may have been of importance. Interestingly, one member of a brother pair had a spermatocytic seminoma. The occurrence of this rare tumour in a brother of a TC patient indicates that spermatocytic seminomas, although believed to be different from other germ cell tumours in terms of pathogenesis, may share aetiological factors with the other germ cell tumours.

Our finding of a greater proportion of bilateral tumours in familial as compared with sporadic TC is in agreement with published reports (Dieckmann *et al.*, 1987; Forman *et al.*, 1992). The finding strengthens the argument that bilateral cases may be genetic.

The point estimate of the SIR is greater in the younger generation, the risk to brothers being larger than the risk to fathers. Data are too sparse to allow estimation of risk to sons. The increased SIR in the younger generation may indicate genetic anticipation. This phenomenon has previously been suggested to be present in TC (Raghavan *et al.*, 1980). If such a mechanism was operating, this would give an indication of what sort of predisposition gene might be involved. Our finding that sons had an earlier age of onset than fathers in affected father–son pairs and that the age of onset in the father–uncle generation was higher than in the remaining familial cases supports the anticipation theory. The former also has been observed previously (Forman *et al.*, 1992).

However, our findings may be the result of ascertainment bias possibly in combination with reduced fertility in the parent generation. The latter is known to mimic genetic anticipation. The effect is produced because only patients with late onset are able to reproduce. This would be relevant for all TC patients but especially for patients with non-seminomas in the 1950s–1970s. During this time period, non-seminomas, which occur on average 10 years earlier than the seminomas, had a much worse prognosis than seminomas with respect both to survival and chance of reproduction.

If familial cases are indeed genetically predisposed to TC we would expect them to have an early age of onset compared with sporadic cases according to the two-hit model for tumorigenesis originally proposed by Knudson in 1971. This was found by Forman *et al.* (1992) in TC patients from the UK when excluding the older generation to correct for ascertainment bias. In the present data set we found the age difference to be present only in seminomas. In non-seminomas, the familial cases had a higher age at orchiectomy than did the sporadic cases. If our finding of no difference in age of onset is correct, it may imply that the genetic factor(s) of importance, at least for non-seminomas, does not follow the Knudson two-hit model and thus that the gene(s) involved is not a classical tumour-suppressor gene. However, there may be other explanations for our findings. In particular, since TC affects young men who have not completed their reproductive period, complex mechanisms leading to ascertainment bias may be present.

The prevalence of UDT in the familial cases was not different from that of the sporadic cases. Taken together with data from the literature (Forman *et al.*, 1992; Tollerud *et al.*, 1985), this suggests that the risk from being related to a TC patient is not dependent on the risk conferred by UDT.

**Acknowledgements**
This study was supported by the Norwegian Cancer Society. KH is a fellow of the Norwegian Cancer Society.

### References

BORRESEN AL, ANDERSEN TI, TRETLI S, HEIBERG A AND MOLLER P. (1990). Breast cancer and other cancers in Norwegian families with ataxia-telangiectasia. *Genes Chrom. Cancer*, **2**, 339–340.

DIECKMANN KP, BECKER T, JONAS D AND BAUER HW. (1987). Inheritance and testicular cancer. Arguments based on a report of 3 cases and a review of the literature. *Oncology*, **44**, 367–377.

DIXON WJ, BROWN MB, ENGELMAN L AND JENRICH RI. (1990). *BMDP Statistical Software Manual*. University of California Press: Berkeley.

FORMAN D, OLIVER RT, BRETT AR, MARSH SG, MOSES JH, BODMER JG, CHILVERS CE AND PIKE MC. (1992). Familial testicular cancer: a report of the UK family register, estimation of risk and an HLA class 1 sib-pair analysis. *Br. J. Cancer*, **65**, 255–262.

KHOURY MJ, BEATY TH AND LIANG KY. (1988). Can familial aggregation of disease be explained by familial aggregation of environmental risk factors? *Am. J. Epidemiol.*, **127**, 674–683.

KNUDSON AGJ. (1971). Mutation and cancer: statistical study of retinoblastoma. *Proc. Natl Acad. Sci. USA*, **68**, 820–823.

RAGHAVAN D, JELIHOVSKY T AND FOX RM. (1980). Father-son testicular malignancy. Does genetic anticipation occur? *Cancer*, **45**, 1005–1009.

TOLLERUD DJ, BLATTNER WA, FRASER MC, BROWN LM, POTTERN L, SHAPIRO E, KIRKEMO A, SHAWKER TH, JAVADPOUR N, O'CONNELL K, STUTZMAN RE AND FRAUMENI JR JF. (1985). Familial testicular cancer and urogenital developmental anomalies. *Cancer*, **55**, 1849–1854.